# Epigenetic Alterations in a Gastric Leiomyoma

**DOI:** 10.1155/2014/371638

**Published:** 2014-12-04

**Authors:** M. T. Branham, M. Pellicer, E. Campoy, M. Palma, A. Correa, M. Roqué

**Affiliations:** ^1^Institute of Histology and Embryology (IHEM-CCT-CONICET) and School of Medical Sciences, National University of Cuyo, M5502JMA Mendoza, Argentina; ^2^Pathology Laboratory, J5402EQC San Juan, Argentina; ^3^Surgery Unit, San Martin Hospital, E3100 Entre Ríos, Argentina; ^4^Surgery Unit, Español Hospital, M5501 Mendoza, Argentina

## Abstract

Leiomyomas constitute 2.5% of all resected neoplasms of the stomach. They are usually asymptomatic, but may present mucosal ulceration. Aberrant DNA methylation is a well-defined epigenetic change in human neoplasms; however, gene-acquired methylation may not necessarily be related with a malignant phenotype. In this report we analyzed in a gastric leiomyoma, the methylation status of 84 CpGI in tumor suppressor and DNA repair genes. We analyzed the tumor center (TC) and tumor periphery (TP) separately. We found aberrant methylation in 2/84 CpGI in the TC portion, that is, *MLH1* and *MSH3*, and 5/84 CpGI in the TP, that is, *MLH1*, *MSH3*, *APC*, *MSH6*, and *MGMT*. The gene with the highest methylation percentage in the TC and TP was *MLH1*. Given that *MLH1* methylation has been associated with microsatellite instability, we analyzed the status of the microsatellite Bat-26. We found that neither the TC nor the TP presented instability. The methylation of *MLH1*, *MGMT*, and *APC* has been described in GISTs, but to the best of our knowledge this is the first time that the methylation of these genes has been associated with gastric leiomyoma. Further research should be conducted to identify reliable molecular markers that could differentiate between GISTs and gastric leiomyomas.

## 1. Introduction

Gastric leiomyomas account for 2.5% of gastric neoplasms. Although most of them are asymptomatic, patients may present upper gastrointestinal hemorrhage [1, 2]. Endoscopically, gastric leiomyomas appear as a large submucosal lesion, and generally endoscopic biopsies are not deep enough to be of any diagnostic value [3]. Pathologically, most of these tumors are composed of spindle cells and display smooth muscle differentiation. Leiomyomas are defined as being desmin and actin positive and c-Kit (or CD117) negative tumors [4].

Tumorigenesis is the result of a multistep process characterized by the accumulation of genetic and epigenetic alterations leading to uncontrolled growth. Among epigenetic modifications, the most studied event in human neoplasms is the deregulation of methylation of DNA, giving rise to widespread changes in the methylome patterns during tumor progression [5]. The epigenome of tumors is characterized by global DNA hypomethylation and by gene-specific hypermethylation. Gene silencing by CpG islands (CpGI) hypermethylation in gene promoters can modulate pathways that control the basic function of the cell by acting directly on tumor suppressor genes and caretaker genes and indirectly on oncogenes through their regulators [5].

Gene expression profile studies have demonstrated that some genes are hypermethylated in gastric GISTs (gastro intestinal stromal tumors) [6–8], but, to our knowledge, there is no information of the methylation profile of gastric leiomyomas.

The objective of this study was to analyze by methyl specific-multiplex ligation probe amplification (MS-MLPA) the methylation status of tumor suppressor and DNA repair genes in a gastric leiomyoma.

## 2. Case Report

A 63-year-old Hispanic female presented with history of melena for two days. Upon clinical examination the patient mentioned that she was treated for* H. pylori* infection 15 years before. No other constitutional symptoms were present. Upper gastrointestinal endoscopy was performed, showing an ulcerated submucosal tumor localized on the cardial region. Upper endoscopic ultrasonography (EUS) evidenced a submucosal lesion, of about 50 mm with decreased echogenicity and homogeneous structure, and no necrotic areas. The lesion belonged to the muscular layer, and EUS suggested GIST (gastrointestinal stromal tumor). Multisided tomography revealed that the lesion was 5 cm and showed no evidence of other lesions in the abdominal area. The patient was scheduled for laparoscopic surgery and was discharged from the hospital eleven days after the operation.

### 2.1. Histopathology

**Figure 1:**
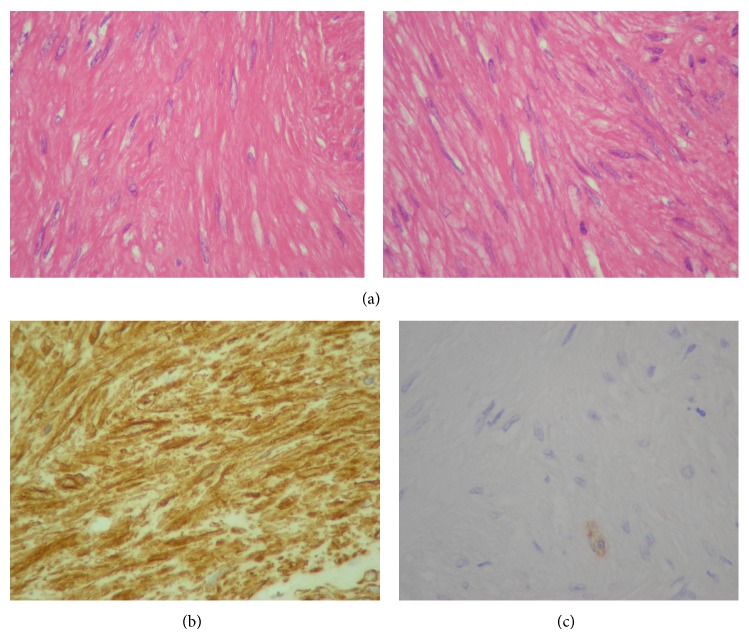
Histopathological images of the gastric leiomyoma analyzed. (a) Intersecting bundles of bland spindle cells in TC (left) and in TP (right). (b) Strong smooth muscle actin immunoreactivity. (c) Negative immunostain with CD117 (a positive mastocyte is shown as positive internal control).

The histopathological diagnosis for the submucosal lesion was gastric leiomyoma, characterized by a proliferation of bland, spindle-shaped cells with elongated nuclei and eosinophilic fibrillary cytoplasm without necrosis and atypia (Figure 1). The mitotic index (number of mitoses per 50 high-power fields, HPF) was <2/50 HPF. The immunohistochemistry assay indicated diffuse positivity for vimentin, smooth muscle actin, and desmin and negative staining for c-Kit/CD117, CD34, cytokeratins AE1/AE2, and S-100. The morphology of the lesion along with the immunohistochemical features supported the diagnosis of leiomyoma.

### 2.2. Methylation Analysis

**Figure 2:**
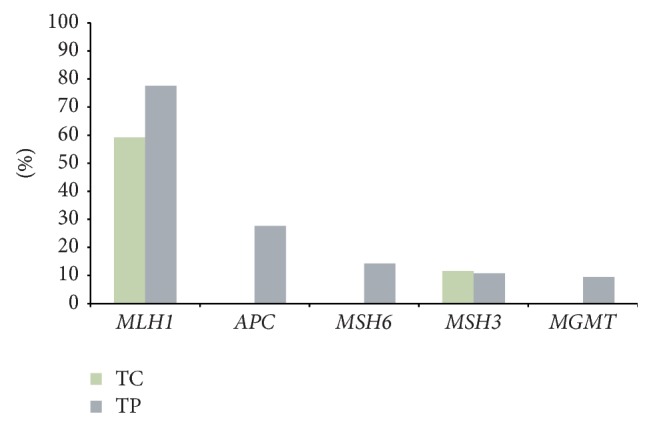
Genes methylated in a gastric leiomyoma. Grey bars represent the genes methylated in the tumor periphery and green bars represent the genes methylated in the tumor center.

MS-MLPA assay was performed by kits ME001, ME002, ME003, ME0024, and ME011 according to manufacturer's, MRC-Holland, Amsterdam, The Netherlands, instructions (http://www.mlpa.com/) on DNA obtained from the formalin fixed embedded tumor. The methylation status of 84 CpGIs in 41 cancer related genes was assessed (Table 1). We have established a cut-off threshold by considering a region to show methylation if the dosage ratio was >8% [9]. Due to tumor heterogeneity and in order to analyze if there were epigenetic differences between different regions of the tumor, we analyzed separately the tumor center (TC) and the tumor periphery (TP). We found aberrant methylation in 2/84 CpGI in the TC portion, that is, one site localized at 485 nt before the transcription start site (TSS) of* MSH3* and one site at 382 nt before TSS of* MLH1*. Even though both genes presented methylation percentages above the established cut-off level, it is interesting to mention that they differed significantly: 11.6% and 52.9%, respectively (Figure 2, green bars). The peripheric portion of the tumor presented a different profile: 5/84 CpG sites were aberrantly methylated, from which 2 sites were shared with the center portion, that is,* MSH3* and* MLH1*, and 3 sites were exclusively methylated in the tumor periphery, that is,* MSH6*,* MGMT*, and* APC* (Table 2). As a control, MS-MLPA assay was performed in normal tissue (i.e., leucocytes of healthy patients); we determined that there was no aberrant methylation in the regions analyzed. The percentages of methylation in the TP were 10.8%, 77%, 14.3%, 9.5%, and 27.7%, respectively (Figure 2, grey bars). Even though the methylated genes differed between the TC and the TP, it is interesting to mention that* MLH1* was shared by both and presented the highest percentage of methylation (52.9% and 77%, resp.). To analyze whether methylation affected gene expression, we performed qRT-PCR assays on 2 cell lines (MDA-MB231 and MCF-7) which presented different percentages of APC promoter methylation (0% and 52%, resp.); we confirmed that the methylation of APC on the CpG site −21 nt before TSS reduces gene expression (data not shown). The methylation of* MLH1* at 382 nt before TSS has been previously shown to provoke downregulation of gene expression [10].

Given that* MLH1* methylation is associated with microsatellite instability (MSI) in sporadic endometrial and colorectal cancers [11, 12], we decided to analyze MSI in the gastric leiomyoma. To evaluate MSI, we analyzed by PCR the status of the mononucleotide microsatellite Bat-26 in the tumor center and periphery. This microsatellite has been shown to be highly efficient and sensitive to determine MSI-H when used as a single marker [13, 14]. Interestingly, Bat-26 was stable in the TC as well as in the TP portion of the gastric leiomyoma analyzed. When we tested Bat-26 status in 5 nontumoral tissues and in peripheral blood of healthy patients, none of these tissues presented Bat-26 instability.

## 3. Discussion

The onset and progression of tumorigenesis involves a cascade of genetic and/or epigenetic events. Results from recent investigations have shown that DNA methylation profiles contain tumor type-specific signatures which, in the future, could serve as biomarkers for clinical outcome [15]. Hypermethylation at the promoter region of several genes has been shown to be an important mechanism in gene silencing. However, gene-acquired methylation may not necessarily be related with malignant phenotype [16, 17]. In this report, we show that a gastric leiomyoma, considered a benign disease, presents methylation in 2–5 of 84 analyzed CpGI, varying in different tumor parts.

The methylation percentage in the overall sample varied widely (from 9.5% to 77%) for the different genes (Figure 1). This wide-ranging methylation levels could be indicating that the aberrant hypermethylation occurs at different sites and times, and, therefore, probably the genes with higher methylation levels (such as* MLH1* or* APC*) were the first ones to be epigenetically altered during tumoral progression. Considering that* MLH1* presents a high methylation percentage, we hypothesize that its methylation could be an initial epigenetic event during gastric leiomyoma formation.

Given that* MLH1* methylation is associated with MSI in several tumors [11, 12], we analyzed the status of the mononucleotide microsatellite Bat-26. As we mentioned before, neither the TC nor the TP portion presented Bat-26 instability. We speculate that the gastric leiomyoma analyzed is not MSI-H due to Bat-26 stability, but we cannot discard MSI-L or MSI-S. It is interesting to mention that the fact that MLH1 is methylated and MSI is stable has been also described by other authors and several hypotheses have been proposed. For example, studies performed by Esteller and colleagues on endometrial atypical hyperplasia concluded that MLH1 promoter methylation is an early event and in some cases may precede a detectable MSI phenotype [18]. Therefore, a possible explanation could be that the leiomyoma presents MLH1 methylation as an early event, lacking yet MSI signs. In another work performed by Kanaya et al. the authors studied the region 700 bp upstream of MLH1 promoter region covering 48 CpG sites; they classified the methylation status as full (over 80% of CpGs were methylated), partial (10–80%), or nonmethylation (less than 10%). The authors concluded that the degree, rather than region-specific methylation of CpG islands, is critical for MSI phenotype [19].

The genes found methylated in the gastric leiomyoma play different functions in the cell:* MLH1*,* MSH3*,* MGMT*, and* MSH6* participate in DNA repair functions whereas* APC* gene participates in cell proliferation. The methylation of* MLH1*,* MGMT*, and* APC* has been previously described in GISTs, but to the best of our knowledge this is the first time that the methylation of these genes has been associated with gastric leiomyoma, which is considered a benign disease. Interestingly, we also found that there were differentially methylated genes between GISTs and the gastric leiomyoma. For example, in a study performed by House et al., the authors determined that the most frequently methylated genes in GISTs (in decreasing frequency) are* MGMT*,* p16*,* RASSF1A*,* E-cadherin*, and* MLH1*  [6]. In another report performed by Saito et al., the authors determined that, besides* MLH1* and* MGMT*, GISTs also presented methylation in* MINT2*,* p73*,* p16*,* E-cadherin*,* MINT1*,* p15*, and* MINT3*. Moreover, the methylation of* RASSF1A* progressively increased from small to malignant GISTs and* p16* was specifically methylated in malignant-prone and malignant GIST [8]. Note that neither* RASSF1*,* p16*, nor* p73* is methylated in the studied gastric leiomyoma. These epigenetic differences between malignant GISTs and gastric leiomyoma have not been described before.

To the best of our knowledge this is the first report showing the methylation profile of a gastric leiomyoma. Further research should be conducted to identify reliable and accurate molecular markers that could help in the differentiation between GISTs and gastric leiomyomas.

## Figures and Tables

**Table 1:** List of genes analyzed in the gastric leiomyoma. Gene names and gene symbols are according to the HGNC database.

Gene symbol	Gene name
APC	Adenomatous polyposis coli
ATM	Ataxia telangiectasia mutated
BCL2	B-cell CLL/lymphoma 2
BNIP3	BCL2/adenovirus E1B 19 kDa interacting protein 3
BRCA1	Breast cancer 1
BRCA2	Breast cancer 2
CACNA1A	Calcium channel, voltage-dependent, P/Q type, alpha 1A subunit
CACNA1G	Calcium channel, voltage-dependent, P/Q type, alpha 1B subunit
CADM1	Cell adhesion molecule 1
CCND2	Cyclin D2
CD44	CD44 molecule
CDH13	Cadherin 13
CDKN1B	Cyclin-dependent kinase inhibitor 1B
CDKN2A	Cyclin-dependent kinase inhibitor 2A
CHFR	Checkpoint with forkhead ring finger domains
CREM	cAMP responsive element modulator
DAPK1	Death-associated protein kinase 1
DLC1	DLC1 Rho GTPase activating protein
ESR1	Estrogen receptor 1
FHIT	Fragile histidine triad
GATA5	GATA binding protein 5
GSTP1	Glutathione S-transferase pi 1
H2AFX	H2A histone family, member X
HIC1	Hypermethylated in cancer 1
HLTF	Helicase-like transcription factor
ID4	Inhibitor of DNA binding 4, dominant negative helix-loop-helix protein
MGMT	O-6-Methylguanine-DNA methyltransferase
MLH1	MutL homolog 1
MLH3	MutL homolog 3
MSH2	MutS homolog 2
MSH3	MutS homolog 3
MSH6	MutS homolog 6
PAH	Phenylalanine hydroxylase
PAX5	Paired box 5
PAX6	Paired box 6
PMS2	PMS2 postmeiotic segregation increased 2
PRDM2	PR domain containing 2, with ZNF domain
PTCH1	Patched 1
PTEN	Phosphatase and tensin homolog
PYCARD	PYD and CARD domain containing
RARB	Retinoic acid receptor beta
RASSF1	Ras association domain family member 1A
RB1	Retinoblastoma 1
RUNX3	Runt-related transcription factor 3
SCGB3A1	Secretoglobin, family 3A, member 1
SFRP4	Secreted frizzled-related protein 4
SFRP5	Secreted frizzled-related protein 5
STK11	Serine/threonine kinase 11
TGIF1	TGFB-induced factor homeobox 1
THBS1	Thrombospondin 1
TIMP3	TIMP metallopeptidase inhibitor 3
TP53	Tumor protein p53
TP73	Tumor protein p73
TWIST1	Twist family bHLH transcription factor 1
VHL	Von Hippel-Lindau tumor suppressor
WT1	Wilms tumor 1

**Table 2:** List of methylated genes in the gastric leiomyoma studied. Gene names and gene symbols are according to the HGNC database; CpG site locations are mentioned based on the MRC-Holland data sheets. TP indicates tumor periphery and TC tumor center.

Gene symbol	Name	CpG location	Sample	Mehtylation %
APC	Adenomatous polyposis coli	21 nt before TSS	TP	27.7
			TC	0

MGMT	O-6-Methylguanine-DNA Methyltransferase	233 nt after TSS	TP	9.5
			TC	0

MSH6	mutS homolog 6	317 nt before TSS	TP	14.3
			TC	0

MLH1	MutL Homolog 1	382 nt before TSS	TP	77.6
			TC	59.2

MSH3	mutS homolog 3	485 nt before TSS	TP	10.8
			TC	11.6

TSS: transcription start site. nt: nucleotide.
